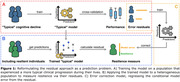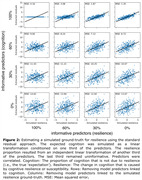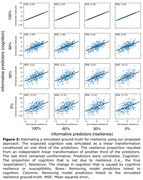# Rethinking the residual approach for estimating cognitive resilience and resistance to AD pathology

**DOI:** 10.1002/alz.094824

**Published:** 2025-01-09

**Authors:** Colin Birkenbihl, Madison Cuppels, Rory Boyle, Hannah M Klinger, Oliver Langford, Gillian T Coughlan, Jasmeer P. Chhatwal, Julie C Price, Aaron P Schultz, Michael J Properzi, Dorene M Rentz, Rebecca E Amariglio, Keith A Johnson, Rebecca F. Gottesman, Shubhabrata Mukherjee, Paul Maruff, Yen Ying Lim, Colin L Masters, Alexa S Beiser, Susan M. Resnick, Samantha C. Burnham, Ilke Tunali, Susan M. Landau, Ann D Cohen, Sterling C. Johnson, Tobey J. Betthauser, Sudha Seshadri, Samuel N. Lockhart, Sid E. O'Bryant, Prashanthi Vemuri, Reisa A Sperling, Timothy J. Hohman, Michael C. Donohue, Rachel F Buckley

**Affiliations:** ^1^ Massachusetts General Hospital, Harvard Medical School, Boston, MA USA; ^2^ Penn Frontotemporal Degeneration Center, Department of Neurology, Perelman School of Medicine, University of Pennsylvania, Philadelphia, PA USA; ^3^ University of Pennsylvania, Philadelphia, PA USA; ^4^ University of Southern California, San Diego, CA USA; ^5^ National Institute of Neurological Disorders & Stroke Intramural Research Program, National Institute of Health, Bethesda, MD USA; ^6^ University of Washington, School of Medicine, Seattle, WA USA; ^7^ Cogstate Ltd., Melbourne, VIC Australia; ^8^ Turner Institute for Brain and Mental Health, School of Psychological Sciences, Monash University, Melbourne, VIC Australia; ^9^ The Florey Institute of Neuroscience and Mental Health, University of Melbourne, Melbourne, VIC Australia; ^10^ Boston University School of Medicine, Boston, MA USA; ^11^ National Institute on Aging, National Institutes of Health, Baltimore, MD USA; ^12^ Eli Lilly and Company, Indianapolis, IN USA; ^13^ University of California, Berkeley, Berkeley, CA USA; ^14^ University of Pittsburgh Alzheimer’s Disease Research Center (ADRC), Pittsburgh, PA USA; ^15^ Wisconsin Alzheimer’s Disease Research Center, University of Wisconsin School of Medicine and Public Health, Madison, WI USA; ^16^ University of Wisconsin School of Medicine and Public Health, Madison, WI USA; ^17^ University of Texas Health San Antonio, San Antonio, TX USA; ^18^ Wake Forest School of Medicine, Winston‐Salem, NC USA; ^19^ University of North Texas Health Science Center, Fort Worth, TX USA; ^20^ Mayo Clinic, Rochester, MN USA; ^21^ Department of Neurology, Vanderbilt University Medical Center, Nashville, TN USA

## Abstract

**Background:**

The residual approach has found wide application in researching cognitive resilience, a phenomenon conceptually understood as cognitive performance being better‐than‐typical for an individual, despite apparent AD pathology. The standard residual approach extracts information about an individual’s resilience from the residuals of a linear model predicting cognition. This approach is subject to several limiting assumptions. Here, we explore the implications of these assumptions and propose an alternative predictive modeling approach which circumvents them.

**Methods:**

Our alternative approach relies on training a machine learning model on a sample of participants who follow a “typical” clinical progression (Fig. 1A). Residuals are then extracted for a heterogeneous dataset that potentially includes resilient participants by comparing observed cognitive outcomes to those predicted by the “typical” model (Fig. 1B). Additionally, our estimate can be refined by effectively estimating and removing model error from the extracted residual by leveraging a second predictive model (Fig. 1C). Using simulated data, we explore the assumptions made by the standard approach and the consequences of their violation. We then contrast this against our approach.

**Results:**

Our simulations demonstrated that the standard residual approach only extracted accurate resilience estimates if the predictors used in the model explained cognition sufficiently well, while being unrelated to resilience itself. That is, as the predictors explain more of the resilience, the residuals became increasingly determined by model error (Fig. 2). We found that residuals achieved with our approach are more precise and provide individualized estimates that are unaffected by the aforementioned trade‐off (Fig. 3).

**Conclusions:**

Our approach holds several advantages over the standard approach: The definition of resilience can be freely adjusted and enforced via different definitions of “typical”, it allows for incorporating complex, non‐linear models without removing relevant information from the residual, the error component of the residual can be estimated through cross‐validation, and all predictors that potentially explain cognition can be included into the model without losing resilience information from the residual. Our approach can be used to measure any phenomenon that can be operationalized as a deviation from a typical outcome and, thus, is also applicable for researching resistance to pathology.